# Biomechanical effects of attachment designs in maxillary first molar mesialization with clear aligners: a finite element study

**DOI:** 10.1186/s40510-025-00578-y

**Published:** 2025-09-30

**Authors:** Resul Vatansever, Hakan Gurcan Gurel, Osman Koc

**Affiliations:** 1 Biruni University, Istanbul, Türkiye; 2 Yıldız Technical University, Istanbul, Türkiye

**Keywords:** Clear aligners, Finite element analysis, Molar mesialization, Orthodontic attachments, Biomechanics

## Abstract

**Background:**

Clear aligners face biomechanical limitations in complex tooth movements, particularly first molar mesialization, despite attachment use. Finite element analysis (FEA) can elucidate optimal attachment designs for force delivery.

**Objectives:**

To compare displacement patterns and stress distribution during maxillary first molar mesialization using four attachment designs via 3D FEA.

**Methods:**

A maxillary model (MRI-derived) was created with periodontal ligament (PDL), alveolar bone, and clear aligner (0.75 mm thickness). Five scenarios were simulated: no attachment (Model-I), vertical rectangular (Model-II), horizontal rectangular (Model-III), optimized double semi-ellipsoidal (Model-IV), and Yin-Yang attachments (Model-V). Mesial displacement (0.5 mm) was applied, and deformation/stress were analyzed using ANSYS Workbench.

**Results:**

Model-IV (optimized attachment) demonstrated the least first molar tipping, evidenced by its lowest total mesial crown displacement (0.319 mm), and provided the best mesiodistal control. Conversely, Model-I (no attachment) exhibited the highest tipping, with a total mesial crown displacement of 0.376 mm. Yin-Yang attachments (Model-V) significantly reduced buccal displacement by 92% compared to Model-I. Horizontal rectangular attachments minimized rotational movement.

**Conclusions:**

Optimized double semi-ellipsoidal attachments provide superior first molar mesialization control, while Yin-Yang designs enhance buccal-lingual stability. Attachment geometry critically influences aligner efficacy.

**Supplementary Information:**

The online version contains supplementary material available at 10.1186/s40510-025-00578-y.

## Introduction

The use of clear aligners in orthodontic treatment has become widespread due to their advantages such as aesthetics, comfort, and improved oral hygiene [1, 2]. However, their biomechanical properties differ significantly from fixed appliances. Being made of viscoelastic polymer materials, clear aligners may not always transmit sufficient force, often leading to the need for additional corrections at the end of treatment [3].

While clear aligners provide successful results in movements such as diastema closure, arch widening, and distalization, procedures such as lateral and premolar rotation, deep bite corrections, molar mesialization, and root movement are often difficult [4–6].

The forces exerted by clear aligners can diminish over time due to factors such as friction, force degradation, and variations in tooth size and shape. Consequently, the aligners may not maintain optimal engagement with the teeth, leading to insufficient desired movement and potential treatment failure [7, 8]. To address this challenge, composite attachments are bonded to the tooth surface to facilitate the creation of an appropriate force system. While numerous attachment types have been developed for various tooth movements, the most mechanically effective design remains largely unclear [9].

Several finite element analyses (FEA) have been conducted to evaluate the effects of attachments on tooth movement [10–14]. For instance, one study investigated a crescent-shaped attachment for incisor body movement and reported favorable outcomes. However, this and similar studies often lack comprehensive information regarding the effectiveness of other attachment types [14].

Therefore, this study aimed to investigate the biomechanical effects of different attachment types, particularly in challenging and complex movements such as maxillary first molar mesialization, using finite element analysis.

## Materials and methods

As part of the BodyParts project, the maxillary and dental model of a young adult male skull obtained by magnetic resonance imaging (MRI) was downloaded as open source in STL format [15].

Using Ansys Workbench, Spaceclaim (ANSYS Inc. Houston, PA, USA) software, a 0.25 mm thick periodontal ligament (PDL) was created around the tooth geometry. A cortical bone layer with a uniform thickness of 2 mm was then modeled, with the alveolar bone defined internally. The PDL volume was established by subtracting the tooth and bone solid models from a larger encompassing volume. As the scenario models focused solely on the mesialization of the maxillary first molar, the second premolar was excluded from the model to streamline the analysis. Attachments were placed specifically on the first molar.

To compare the effects of models without attachments and with different attachment geometries, five distinct clear aligner models were generated. The clear aligner geometry for each scenario was designed using 3Shape digital design software (3Shape, Copenhagen, Denmark), with a thickness of 0.75 mm. This thickness was chosen as it is commonly adopted in similar research [16–18]. The aligners were designed to be fully compatible with the tooth crown surface and to precisely wrap the attachments.

Four distinct attachment types were designed for the simulation scenarios: vertical rectangular (4 × 2 × 1.5 mm), horizontal rectangular (2 × 4 × 1.5 mm), optimized double semi-ellipsoidal (r:1.4), and a novel Yin-Yang attachment. The Yin-Yang attachment was designed with two inversely symmetrical halves: a mesial half (2.5 mm wide horizontal rectangle, gingivally inclined, 1.75 mm thick, with a 0.5 mm prominence directed towards the crown surface) and a distal half (similarly inclined and tapered 2.5 mm wide horizontal rectangle, 1.75 mm thick, with a 0.5 mm prominence directed towards the crown surface). The geometry of the Yin-Yang attachment was digitally modeled based on its conceptual design. In all five aligner models (including the no-attachment control), the clear aligner geometry featured a 0.5 mm gap in the mesial aspect of the molar tooth to facilitate mesial movement of the first molar.

**Fig. 1:**
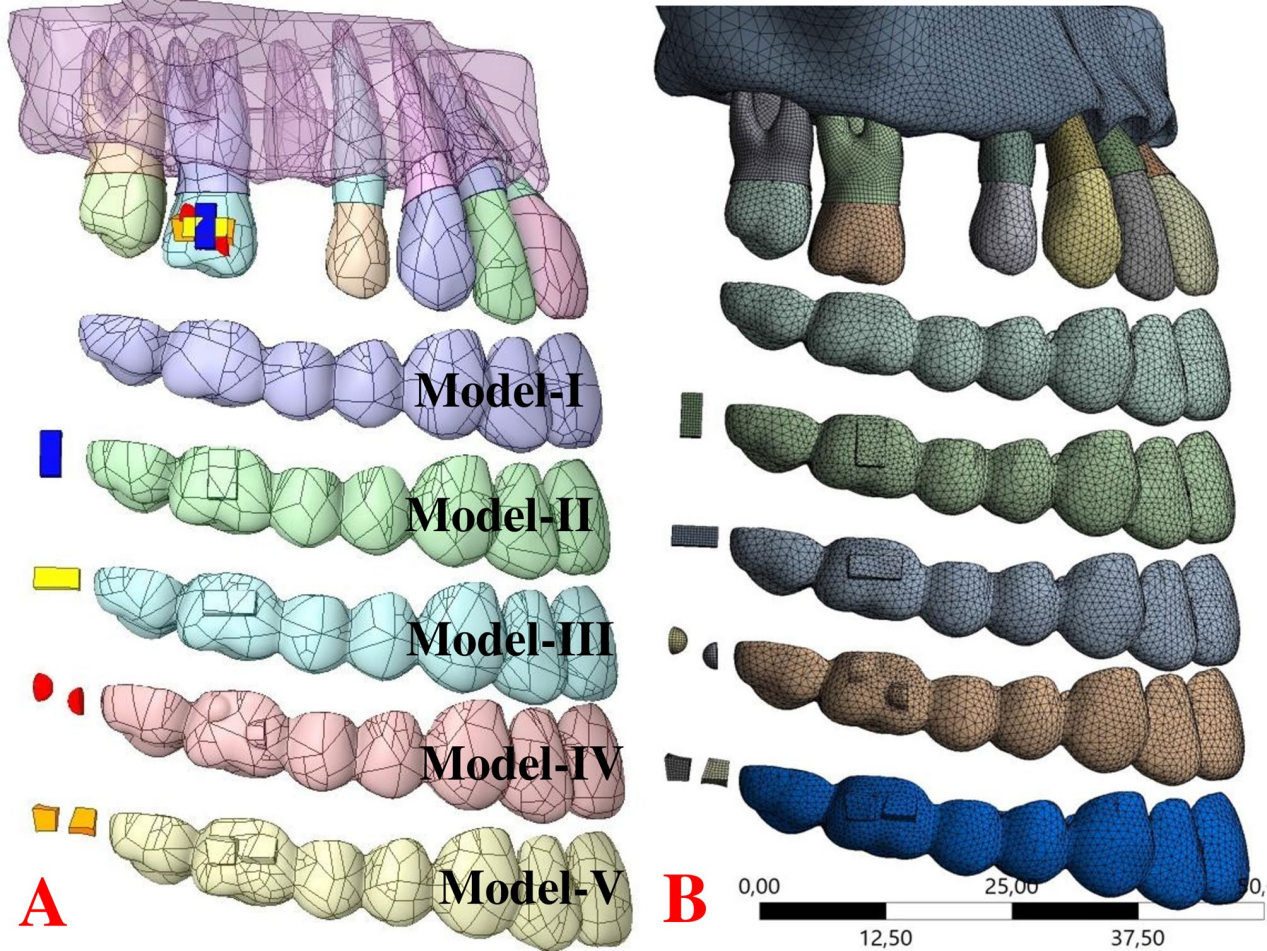
**A**: Illustration of the five different FEA simulation scenario models created for maxillary first molar mesialization: (I) clear aligner model without attachment, (II) with vertical rectangular attachment, (III) with horizontal rectangular attachment, (IV) with optimized double semi-ellipsoidal attachment, and (V) with Yin-Yang attachment. **B** Illustration of the detailed mesh structure and refinement applied to the symmetric scenario model, including the maxilla, clear aligner, teeth, periodontal ligament (PDL), and attachments

All components, including the maxilla (cortical and alveolar bone), tooth, periodontal ligament, attachments, and clear aligners, were assembled independently for each of the five scenario models within the same coordinate system (Fig. 1A).

### Mesh structure

The symmetric scenario model was discretized into a total of 344,186 nodes and 206,509 elements. Specifically, the maxilla was meshed with 1.6 mm elements. The clear aligner, teeth, and periodontal ligament (PDL) model were meshed using 0.8 mm 10-node quadratic tetrahedral elements, while the molar PDL utilized 0.4 mm elements. The attachments were discretized with 0.3 mm 20-node quadratic hexahedral elements (Fig. 1.B). To ensure mesh convergence and improve accuracy, element sizes were refined to 1 mm in specific regions of the maxillary surface and to 0.4 mm in the area where the attachment interfaces with the clear aligner.

The element and node counts, along with the mesh element sizes, were approximately consistent across all models. The average mesh skewness value, a key indicator of element quality, was 0.25 (Table 1). The quality and convergence of the mesh for the model’s geometric parts were evaluated using the ANSYS software. The average values for both metrics were determined to be ‘excellent’ according to the mesh metrics spectrum. This mesh element quality metric (mesh skewness spectrum) is a commonly reported parameter in previous FEA studies [19–24]. Table 1 provides a comprehensive overview of the number of nodes and elements, mesh size values, and the element quality range for all anatomical parts of the entire model.

**Table 1:** Finite element model: elements, nodes, mesh size, average skewness, element quality, and convergence values

	Average skewness value	Nodes	Element	Mesh size (millimeters)	Mesh element type
Maxillary bone	0.22	116,019	78,076	1.6	Tetrahedral
Periodontal ligament	0.6	23,887	11,760	0.8	Tetrahedral
Tooth	0.18	104,334	69,236	0.8	Tetrahedral
Molars PDL	0.55	59,388	20,758	0.4	Hexahedral
Clear Aligner	0.36	38,154	20,234	0.8	Tetrahedral
Attachment	0.45	2303	547	0.3	Hexahedral

### Material properties

The cortical and alveolar (cancellous) bone and periodontal ligament (PDL) within the maxilla were considered anisotropic. Conversely, the tooth, attachment, and clear aligner were modeled as homogeneous and isotropic materials. Nonlinear material properties were assigned to both the maxilla and PDL models.

A hyperelastic (third-order Ogden) model was defined as the material for the PDL model of all teeth.

The specific material properties, including Young’s modulus, Poisson’s ratio, shear modulus, and Ogden parameters for nonlinear models, describing each of the aforementioned components are listed in Table 2 [19–25].

**Table 2:** Material properties of finite element model

Material properties (MPa)	Elastic modulus	Poisson’s ratio	Shear modulus
Cortical maxillary bone	Ex: 12,000	Vxy: 0.18	Gxy: 3600
	Ey: 11,640	Vyz: 0.40	Gyz: 5400
	Ez: 15,600	Vxz: 0.30	Gxz: 4100
Cancellous maxillary bone	Ex: 1448	Vxy: 0.32	Gxy: 434
	Ey: 1448	Vyz: 0.05	Gyz: 68
	Ez: 210	Vxz: 0.05	Gxz: 68
Periodontal ligament	µ_1_: − 3420.83	µ_2_: 1434.35	µ_3_: − 5.56E-04
	α_1_: − 0.506	α_2_: − 0.134	α_3_: 13.708
	D_1_: 0 MPa^− 1^	D_2_: 0 MPa^− 1^	D_3_: 0 MPa^− 1^
Dentin	19,890	0.31	
Clear aligner	528	0.36	
Attachment	12,500	0.36	

MPa: Megapascal, Ex, Ey, Ez: Elastic modulus in three directions, Vxy, Vyz, Vxz: Poisson’s ratio in three directions, Gxy, Gyz, Gxz: Shear modulus in three directions, µ, α, D : Material parameters

### Boundary conditions

The coordinate system was defined with the x-axis representing the bucco-palatal direction, the y-axis representing the mesio-distal/anteroposterior direction, and the z-axis representing the longitudinal/vertical (superior-inferior) direction.

In all scenarios, the effect of two clear aligners on the first molar’s mesialization (total movement of 0.5 mm) was analyzed. To accurately reflect the aligner’s movement and facilitate solution convergence, the displacement was applied incrementally in four steps during the analysis.

In the FE model, two sequential analysis solutions were performed to simulate the effect of the clear aligner (CA) on the mesial movement of the first molar.

In the first analysis, a displacement of 0.5 mm in the distal direction was applied as a boundary condition to the distal surface area of the first molar in contact with the clear aligner. This step allowed for the measurement of the clear aligner’s deformation. In the second analysis, the measured amount of deformation from the clear aligner (specifically in the first molar contact zone) was then applied as a displacement boundary condition to the first molar.

Similarly, the reaction forces between the first molar and the clear aligner, and between the attachment and the clear aligner, were calculated in the first analysis. The force calculated in the second analysis, (in the opposite direction), is defined as the boundary condition.

**Fig. 2:**
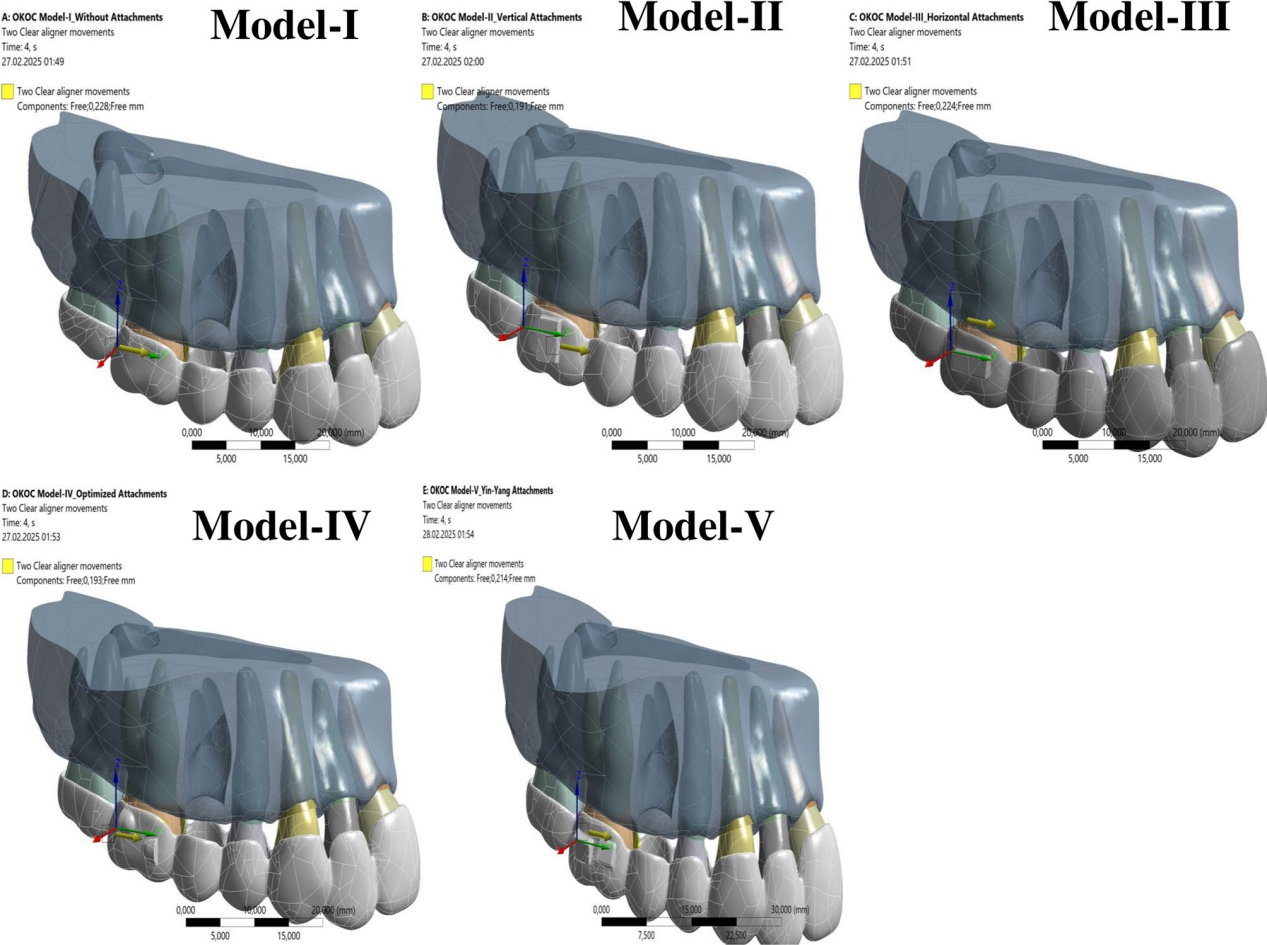
Deformation of the clear aligner from the first analysis, illustrating its application as a displacement boundary condition for the mesial movement of the molar in the second analysis (along the + Y axis, indicated by the yellow arrow)

The calculated amount of movement for each clear aligner was applied as a displacement in the mesial direction (along the + Y axis, as indicated by the yellow arrow in Fig. 2) in the contact region of the first molar with the aligner. The amount of deformation measured for each clear aligner in the mesial direction is shown in Table 3.

**Table 3:** Deformation of the clear aligner in each scenario model from the first analysis solution

	Amount of deformation on the clear aligner (mm)	
	First aligner	Second aligner
Model-I	0.142	0.228
Model-II	0.119	0.191
Model-III	0.129	0.224
Model-IV	0.140	0.193
Model-V	0.137	0.214

### Analysis and evaluation

Displacement values were measured in millimeters (mm) and von Mises stress values in Megapascals (MPa), which together indicate the deformation and stress state of the model.

Geometrical nonlinearity, nonlinear contact theory, and nonlinear material behavior were considered to evaluate the displacement and von Mises stress distribution. A time-dependent structural analysis (dynamic analysis) method was employed to solve the problem. In all scenario models, displacement and stress values were measured pointwise on the same element using a probe within ANSYS Workbench software.

The pterygomaxillary suture region was completely fixed on the model, serving as the primary fixed boundary.

Bonded contact was defined between the teeth and the periodontal ligament, between the teeth and attachments, and between the maxilla and the PDL. In this bonded contact state, the model components moved as a whole, allowing no sliding or separation between the surfaces and edges.

Frictional contact (coefficient of friction µ: 0.2) was defined between the clear aligner and the contact surfaces of the teeth and attachments. These surfaces were allowed to act as individual units, permitting separation or sliding between them [25].

## Results

### Total amount of displacement

Across all scenario models, the total displacement of the first molar resulting from the mesial force applied by two clear aligners was measured over four incremental steps during the analysis. For all models, the greatest displacement was observed at the tip of the molar cusps, while the smallest displacement was found in the distal region of the palatal root near the apex.

**Fig. 3:**
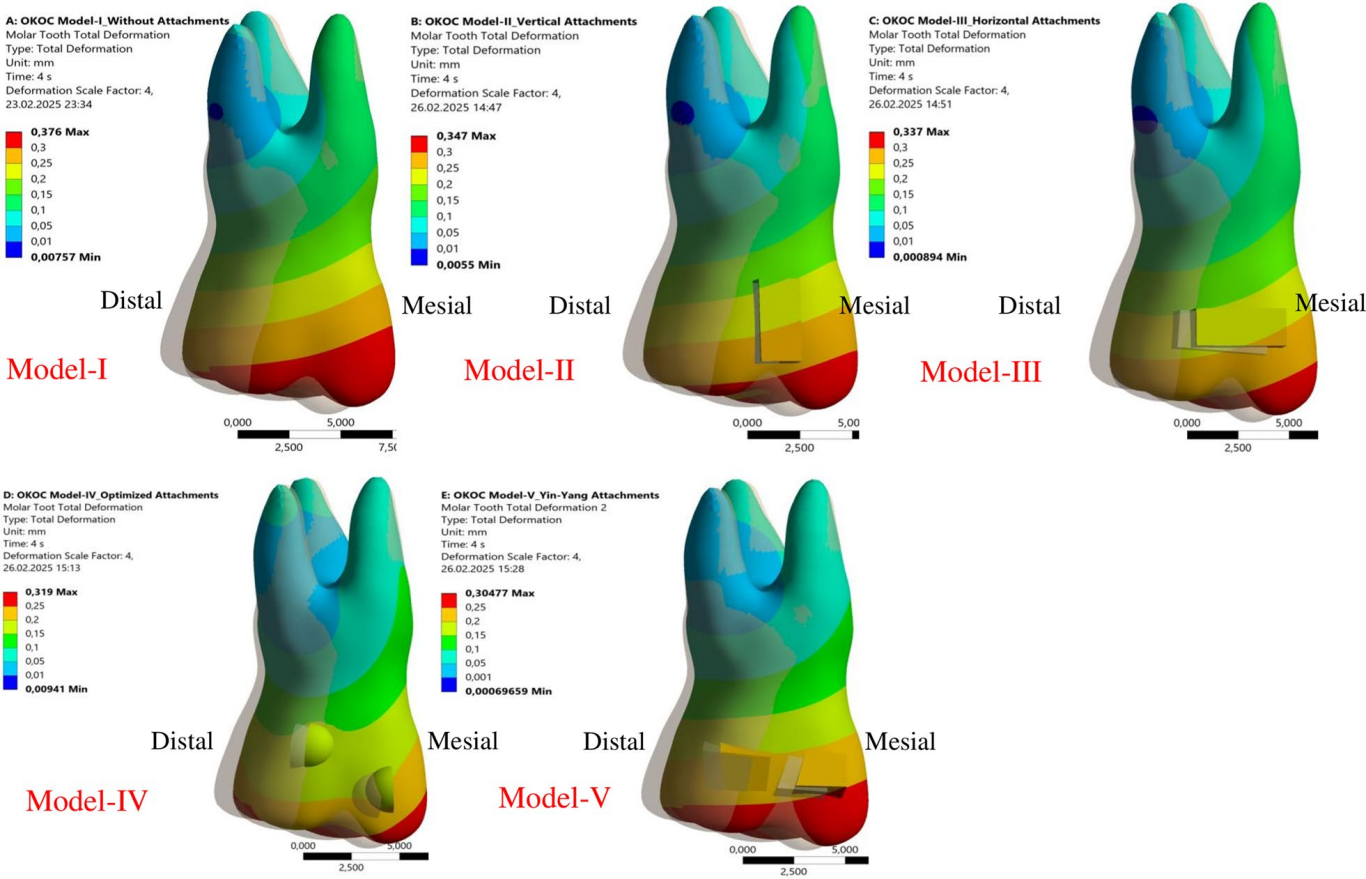
Maximum total mesial displacement (mm) across models (scale 4X)

Following the first aligner movement, the maximum total displacement on the molar was located at the mesiobuccal cusp tip in Model I, and at the distobuccal cusp tip in the other four models. The highest total displacement of the first molar was recorded in Model IV (0.242 mm), and the lowest in Model I (0.179 mm) (Table 4; Fig. 3).

Following the second aligner movement, the location of maximum total displacement varied: it was observed at all cusps in Model I, at the mesiobuccal and mesiopalatal cusps in Models II and III, at the mesiopalatal cusp in Model IV, and at the mesiobuccal cusp in Model V.

The maximum total displacement in the mesial direction at the conclusion of the analysis was measured as follows: Model I (0.376 mm), Model II (0.347 mm), Model III (0.337 mm), Model IV (0.319 mm), and Model V (0.304 mm) (Table 4; Fig. 3).

**Table 4:** Total and directional displacement (mm) of the molar tooth in five scenario models after two sequential clear aligner movements

Model scenarios clear aligner	Molar tooth movement (mm)								
	Total displacement mm			Buccal/lingual direction (X) mm		Mesial/distal direction (Y) mm		Apical/coronal direction (Z) mm	
Clear aligner movements		1st Aligner	2nd Aligner	1st Aligner	2nd Aligner	1st Aligner	2nd Aligner	1st Aligner	2nd Aligner
Model-I_Without Attachment	Max	**0.179**	**0.376**	**0.013**	**0.037**	**0.171**	**0.358**	**0.075**	**0.156**
	Min	0.001	0.007	− 0.008	− 0.022	− 0.045	− 0.090	− 0.041	− 0.076
Model-II_Vertical Attachment	Max	**0.206**	**0.347**	**0.060**	**0.022**	**0.201**	**0.332**	**0.067**	**0.141**
	Min	0.007	0.005	− 0.050	− 0.017	− 0.059	− 0.092	− 0.038	− 0.077
Model-III_Horizontal Attachment	Max	**0.215**	**0.337**	**0.041**	**0.014**	**0.208**	**0.321**	**0.078**	**0.141**
	Min	0.010	0.000	− 0.032	− 0.014	− 0.074	− 0.082	− 0.042	− 0.073
Model-IV_Optimized Attachment	Max	**0.242**	**0.319**	**0.063**	**0.053**	**0.237**	**0.320**	**0.071**	**0.110**
	Min	0.005	0.009	− 0.055	− 0.036	− 0.083	− 0.062	− 0.055	− 0.077
Model-V_Yin-Yang Attachment	Max	**0.199**	**0.304**	**0.048**	**0.005**	**0.195**	**0.300**	**0.057**	**0.110**
	Min	0.006	0.000	− 0.050	− 0.025	− 0.075	− 0.068	− 0.046	− 0.046

X-axis: +x buccal, -x lingual; Y-axis: +y mesial, -y distal; Z-axis: +z intrusion, -z extrusion

### Directional displacements

#### Buccal/lingual displacement

After the second aligner movement, the buccal and lingual displacement of the first molar in Model-I (without attachment) was greater compared to its displacement after the first aligner movement. Conversely, in models incorporating attachments, the buccal and lingual displacement of the first molar after the second aligner movement was smaller than that observed after the first aligner movement.

**Fig. 4:**
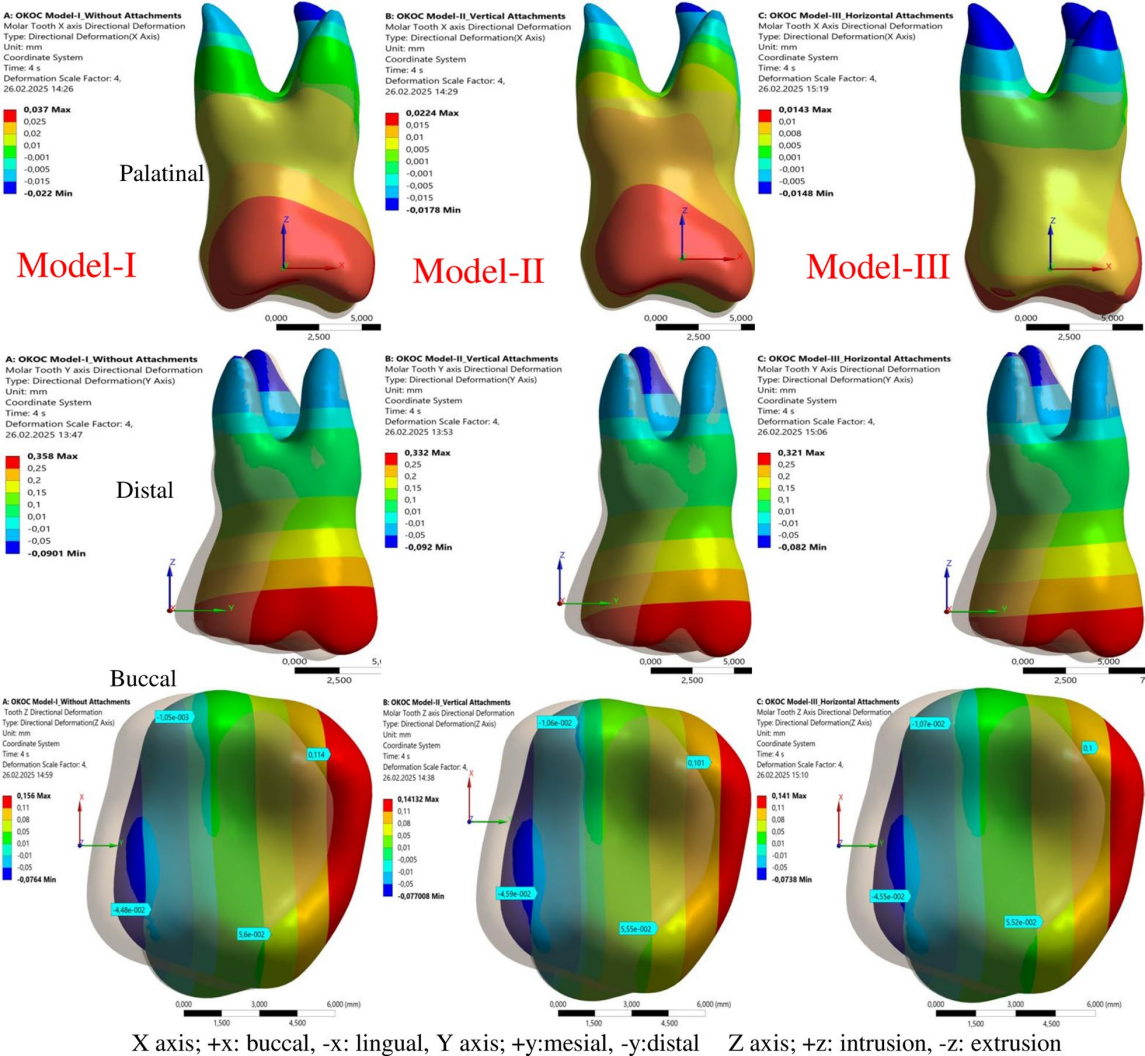
Buccal (+ X) displacement (mm) in Models I, II and III for the second aligner movement (scale 4X)

**Fig. 5:**
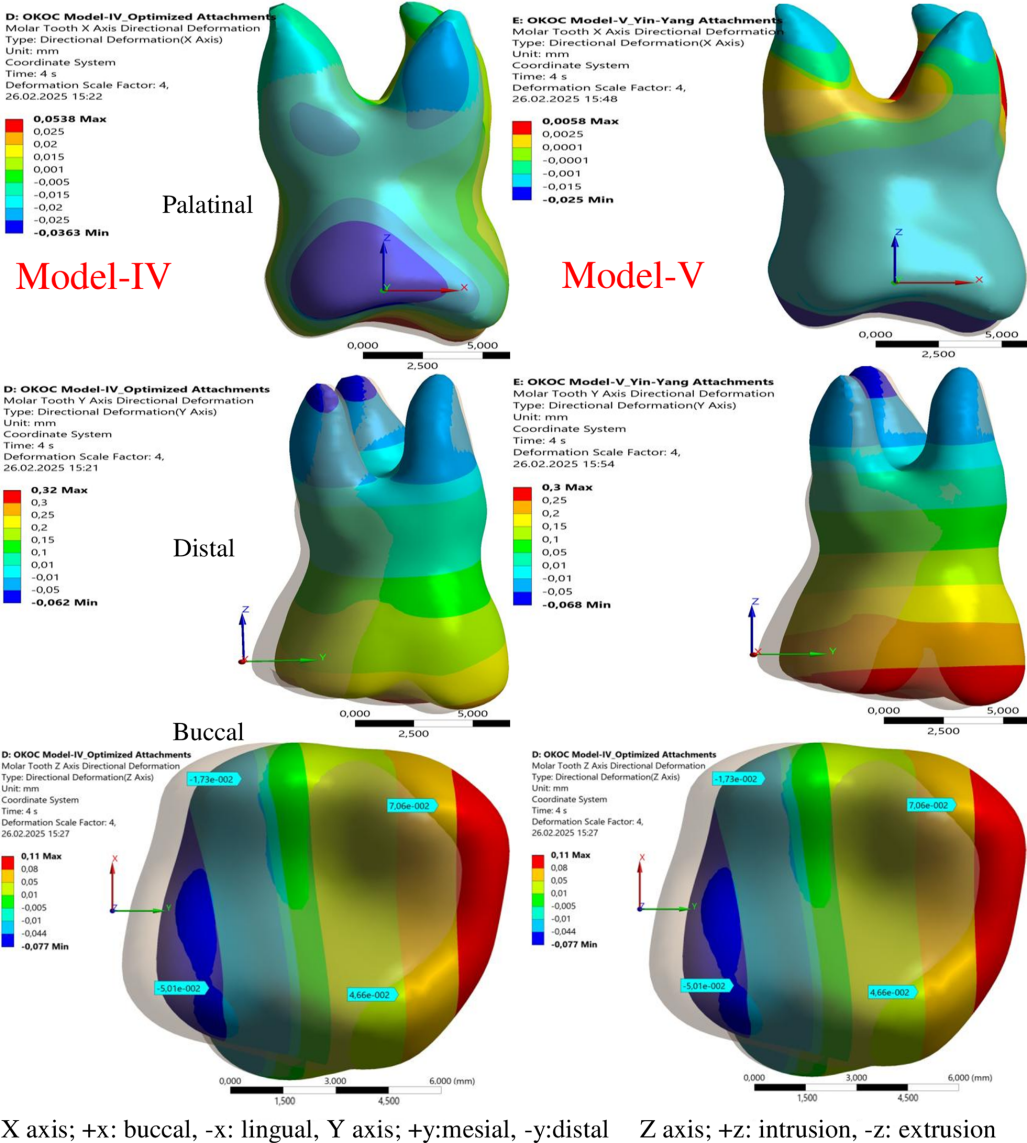
Buccal (+ X) displacement (mm) in Models IV and V for the second aligner movement (scale 4X)

Among all models, the largest maximum displacement measured in the buccal (+ X) direction for the second aligner movement was observed in Model-IV (0.053 mm), while the smallest maximum displacement was found in Model-V (0.005 mm) (Table 4; Figs. 4 and 5).

### *Mesial/distal andapical/coronal displacement*

In all models, the displacement measured in the mesial (+ Y) direction in the molar and the movement in the distal (-Y) direction in the root increased after the second aligner movement. After the first aligner movement, the displacement measured in the mesial (+ Y) direction was the largest in Model-IV (0.237 mm) and the smallest in Model-I (0.171 mm). After the second aligner movement, the largest mesial displacement was measured in Model-I (0.358 mm) and the smallest in Model-V (0.300 mm) (Table 4; Figs. 4 and 5).

After the second aligner movement, the molar displacement in the vertical direction increased and was measured larger in all models compared to the first aligner movement. Among the models, the largest and smallest displacement measured in the molar in the apical (+ Z) direction were found in Model-III (0.078 mm) and Model-V (0.057 mm), respectively, for the first aligner movement. After the second aligner movement, the largest displacement in the apical direction was measured in Model-I (0.156 mm), then the same in Models-II and III (0.141 mm), and the smallest in Models-IV and V (0.110 mm) (Table 4; Figs. 4 and 5).

### Angular changes due to movement

**Table 5:** Molar rotation and tipping angles (°) measured in five scenario models during clear aligner movement

Model scenarios clear aligner	Molar angle of rotation and tipping		
	Buccal tipping (X)	Mesial tipping (Y)	Rotation (Z)
Model-I_Without Attachment	0.7°	4.2°	2.7°
Model-II_Vertical Attachment	0.4°	3.6°	3.5°
Model-III_Horizontal Attachment	0.4°	3.8°	2.7°
Model-IV_Optimized Attachment	0.5°	3.0°	3.0°
Model-V_Yin-Yang Attachment	0.3°	3.8°	4.5°

X-axis: buccolingual angulation; Y-axis: mesiodistal angulation; Z-axis: rotational angulation

In all models, the first molar exhibited angular changes in all three directions following both the first and second aligner movements. After the second aligner movement, the angle of displacement in the buccal (+ X) direction increased. The largest angular change in the buccal direction was observed in Model I (0.7°), while the smallest was 0.3°. Similarly, mesial (+ Y) angular displacement increased after the second movement, with the highest value in Model I (4.2°) and the lowest in Model IV (3.0°). Additionally, rotation around the vertical (+ Z) axis also increased following the second aligner movement, with the greatest angular change found in Model V (4.5°), and the smallest values observed in Model I (2.7°) and Model III (2.7°), which were close to each other (Table 5).

## Discussion

In clear aligner treatment, three-dimensional software is routinely used to plan the final desired tooth movements within the oral cavity. However, a common clinical observation is that these planned movements do not always correspond precisely to the actual clinical outcomes. This discrepancy can be attributed to the fact that computer-generated designs may not fully account for the intricate mechanical properties of clear aligners and the complex biomechanical responses of the teeth during clinical application [26]. Considering the inherent biomechanical factors of the tooth, such as its multirooted anatomy and volumetric size, the judicious selection of attachments plays a crucial role in achieving complex movements like molar mesialization [10]. Furthermore, clinical outcomes can be potentially enhanced by incorporating auxiliary mechanics, such as mini-screws or power arms, alongside clear aligners [18].

However, the implementation of additional mechanics may be constrained by various factors, including increased cost, application difficulties, oral hygiene challenges, and potential patient reluctance or negative perceptions. It is often challenging to achieve root movement with clear aligners alone, particularly in the absence of attachments. In such scenarios, the tooth tends to exhibit more tipping than desired, rather than achieving true bodily movement. For complex movements like mesialization or distalization, the use of attachments becomes essential [13, 27]. This necessity arises because attachments, when strategically placed on the buccal surface of the tooth, generate a counter-moment that supports bodily movement and provides crucial resistance to unwanted tipping forces [9]. Consistent with this, the results of the present study indicate that in the absence of an attachment (Model I), a counterclockwise moment is generated, leading to mesial tilting of the tooth.

The rotational movement observed around the Z-axis is an unplanned effect, and the potential of attachment designs to mitigate this effect is important. This rotation largely stems from imbalanced moment components that arise due to the distance between the tooth’s center of resistance (CR) and the point of force application. Particularly during mesialization, as aligner forces are applied closer to the tooth crown, it is not surprising for the tooth to exhibit rotational movement. Therefore, the extent to which different attachment configurations are effective in controlling this undesirable rotational moment is a clinically critical area of evaluation. In the present study, while the horizontal rectangular attachment best controlled rotational movements, the Yin-Yang and optimized double semi-ellipsoidal attachments also yielded similarly successful results.

The vertical rectangular attachment is widely preferred for clinically challenging tooth movements. However, limited evidence exists to support its effectiveness in molar mesialization or distalization. This limitation is largely due to its inability to generate a sufficient counter-moment on the buccal surface to promote bodily movement, resulting instead in undesirable mesial tipping. In a retrospective study, molars in extraction cases were found to exhibit more mesial tipping than initially predicted [26]. In previous studies, the vertical rectangular attachment typically measured 3 mm in length. In the present study, this dimension was increased to 4 mm in an effort to enhance its effectiveness. However, the findings revealed that the 4 mm vertical rectangular attachment produced results similar to those of other attachment types, with no significant difference observed in the degree of mesial tipping.

The use of a horizontal rectangular attachment has been reported to provide better mesiodistal control than a vertical rectangular attachment in molars [26, 28]. However, another study reported no significant difference between these two attachment types in terms of tension, stress, and displacement tendency during molar distalization [29]. According to the results of the present study, no significant difference was observed between the horizontal and vertical rectangular attachments in terms of mesiodistal control, and similar outcomes were found. Nevertheless, the horizontal rectangular attachment resulted in less rotational movement compared to the vertical rectangular attachment during molar mesialization.

To apply the necessary forces for mesial root inclination, the attachments were designed to be placed on the molars in an inversely symmetrical manner (Yin-Yang configuration). This design aims to replicate the effect of a bracket slot engaged with an archwire, where, during mesial movement, the molars tend to tip, and the bracket slot helps to straighten the roots by controlling the wire [28, 29]. This is particularly relevant for cases requiring molar mesialization exceeding 2 mm, where the force system must create a rotation center within the root-crown to effectively influence the root apex and manage the vertical dimension. The Yin-Yang style twin attachments are designed to best simulate this desired wire-bracket interaction. Specifically, the mesial half of the Yin-Yang attachment is a 2.5 mm wide horizontal rectangle inclined gingivally, with a 1.75 mm thickness at the incisal and a 0.5 mm prominence at the gingival aspect, directed towards the crown surface. The distal half is a similarly inclined and tapered 2.5 mm wide horizontal rectangle, also 1.75 mm thick with a 0.5 mm prominence at its gingival aspect, directed towards the crown surface. These inclined structures are intended to balance occlusal and gingival vertical forces, thereby counteracting the tipping moment during molar mesialization [30, 31]. Although the Yin-Yang attachment had not been previously evaluated in an FEA study, rendering its movement effects unobserved and precluding comparisons with other attachments, the present study found its mesiodistal control comparable to that of the horizontal rectangular attachment. However, the amount of rotation during mesialization was greater than observed with the horizontal rectangular attachment. Furthermore, the production and placement of this attachment within the aligner may be more complex than with standard CAD/CAM systems, potentially necessitating software-specific definitions and precise manufacturing positioning for enhanced clinical applicability.

The optimized double semi-ellipsoidal attachment was designed to enhance the retention of clear aligners on the tooth and to enable more controlled mesiodistal tooth movements. The double structure provides a greater surface area compared to single attachments, allowing the forces applied by the aligner to be transferred to the tooth in a more balanced and controlled manner. When the aligner is first inserted, it causes the tooth to tilt and rotate slightly due to elastic deformation, and the moment-to-force ratio increases as the tooth begins to move. Over time, the tooth uprights, the rotation is reversed, and it progresses toward the desired position in a more controlled manner. The optimized double semi-ellipsoidal attachment is widely used for central incisor and canine with long crowns in both current research and clinical applications [9, 13, 27]. In the present study, the objective was to evaluate the effects of this attachment on molar movement. According to the results, the motion effect of the double semi-ellipsoidal attachment on the molar initially involved tilting and rotation, followed by uprighting and rotational correction—similar to the behavior observed in central incisors and canines. When compared with other attachments, this design produced the least amount of tipping during mesiodistal movement. From a clinical applicability perspective, the attachment’s effectiveness may vary depending on the crown length of the tooth. In molars with short crowns, achieving sufficient contact area can be challenging, which may limit the design’s applicability from both a technical and biomechanical standpoint. This situation may be easier in teeth with longer crowns, where the attachment’s placement and stable integration within the aligner can be more readily achieved.

In various clinical and finite element analysis (FEA) based studies found in the literature, it has been shown that mesial tipping for the upper first molar (U6) can reach a level of approximately 5–6° [18, 26]. Elfouly et al., evaluating natural variations obtained after treatment, reported an average tipping value for this tooth of 6.8° ± 6.5°. Similarly, it was stated that the average rotation in the same tooth is around 4.5°, and that values exceeding the 5° limit can be considered clinically significant [32].

Many real-world problems are inherently nonlinear. Despite this, linear analysis methods are often preferred to simplify and speed up the analysis solution by simplifying boundary conditions. However, when the material enters the elastic region (where the relationship between stress and strain is not linear), these simplifications significantly reduce the accuracy of the solution. In nonlinear analysis, the nonlinear relationship between applied forces and resulting displacements arises from nonlinearity in geometry, boundary conditions, and material properties. When these three effects are considered, the FEA method yields results closer to reality. In the orthodontic literature, only a limited number of FEA studies adopt this comprehensive approach, with mostly linear solution methods being used. In this study, however, the analysis solution was performed by considering all three nonlinear conditions [33].

In FEA studies, correctly determining the model’s boundary conditions (contact type, applied forces, displacement magnitudes), material properties, mesh structure (number of elements and nodes), and mesh convergence is critically important to ensure the reliability of the results and the reproducibility of the analysis. The quality of the model’s mesh structure has a direct impact on the accuracy of the solution. According to the mesh metrics spectrum determined by ANSYS software, mesh convergence is required to produce reliable results. Mesh convergence refers to the sufficient number of elements and nodes that should be present in the model, and mesh size changes do not affect the analysis results. The skewness value range of the mesh metrics spectrum has been shown in previous FEA studies. High orthogonality and low skewness values are recommended to increase analysis accuracy. The accuracy of the analysis results is affected by these values. As the average skewness value progresses from ‘excellent’ to ‘very good’ and ‘good,’ the analysis accuracy decreases. In this study, the average skewness quality and mesh convergence value of the parts forming the model’s geometry were determined to be ‘excellent’ [16–34].

### Limitations and future research

The 0.5 mm displacement simulated in our study may not directly represent clinical situations requiring the closure of a second premolar extraction space, which is typically in the 6–7 mm range. However, this limited displacement allows for a comparative evaluation of the initial phase effects of force systems created by different attachment types. FEA programs cannot model physiological events such as new cell formation or destruction in anatomical structures like bone and PDL that occur clinically (i.e., tooth movement). This method, used for calculating mechanical behaviors, cannot fully simulate complex cellular changes in biological systems. In this analysis, tooth movement (tipping, rotation, and displacement amount) was calculated by neglecting tissue/cell healing due to periodontal ligament compression, depending on the clear aligner and attachment type, and the instantaneous change of this effect was compared. This study is the first to compare the effects of different attachment types on a 0.5 mm aligner-driven displacement. 

After the first aligner movement, the position and shape change (coordinate change in x, y, z axes) in the tooth geometry were not taken into account, and the second aligner geometry was not modeled separately. Although the amount of tooth displacement could be determined in the analysis, it is very difficult to re-model based on the obtained solution outputs. The nonlinear (anisotropic) material property is defined to more accurately describe the actual behavior of bone and PDL and to approach the correct solution result.

However, it is an important question whether the effect of attachment designs will continue similarly throughout the entire treatment process and how this effect might change with increasing displacement. Therefore, further studies are needed on the scalability of the obtained findings and the formal effects of accumulated displacement during treatment. In this context, it should also be evaluated whether small differences observed in the early stages become clinically significant as treatment progresses.

## Conclusions

Based on the findings of this study:

- The first molar without an attachment (Model I) exhibited greater tipping and rotational movements during mesialization, highlighting that the use of attachments significantly improved control over these undesired movements.
- While all investigated attachment types enhanced molar movement control, some degree of undesired tipping and rotational movements was still observed across all attachment-inclusive models.
- Optimized double semi-ellipsoidal attachments resulted in the least mesiodistal tipping of the first molar. Rotational movement was most effectively controlled by the horizontal rectangular attachment, with the optimized double semi-ellipsoidal attachments showing comparable results in this regard.

## Supplementary Information

Below is the link to the electronic supplementary material.

Supplementary Material 1.

Supplementary Material 2.

Supplementary Material 3.

Supplementary Material 4.

Supplementary Material 5.

## Data Availability

No datasets were generated or analysed during the current study.

## Abbreviations

FEA: Finite element analysis
PDL: Periodontal ligament
MRI: Magnetic resonance imaging
